# *Faecalibacterium prausnitzii* A2-165 has a high capacity to induce IL-10 in human and murine dendritic cells and modulates T cell responses

**DOI:** 10.1038/srep18507

**Published:** 2016-01-04

**Authors:** Oriana Rossi, Lisette A. van Berkel, Florian Chain, M. Tanweer Khan, Nico Taverne, Harry Sokol, Sylvia H. Duncan, Harry J. Flint, Hermie J. M. Harmsen, Philippe Langella, Janneke N. Samsom, Jerry M. Wells

**Affiliations:** 1 Host-Microbe Interactomics Group, University of Wageningen, 6708 WD, The Netherlands; 2 Department of Pediatrics, Erasmus Medical Center - Sophia Children’s Hospital, 3015 CE, Rotterdam, The Netherlands; 3 Commensal and Probiotics-Host Interactions Laboratory, UMR 1319 Micalis, INRA, 78352, Jouy-en-Josas, France; 4 UMR 1319 Micalis, AgroparisTech, 78352, Jouy-en-Josas, France; 5 Department of Medical Microbiology, University Medical Center Groningen, 9700 RB, Groningen, The Netherlands; 6 Department of Gastroenterology and Nutrition, Hôpital SaintAntoine and UPMC University of Paris, 75012, Paris, France; 7 Equipe AVENIR Gut Microbiota and Immunity, INSERM U1057/UMR CNRS 7203, Université Pierre et Marie Curie 6, 75005, Paris, France; 8 Microbial Ecology Group, Rowett Institute of Nutrition and Health, University of Aberdeen, AB21 9SB, Aberdeen, United Kingdom

## Abstract

*Faecalibacterium prausnitzii* strain A2-165 was previously reported to have anti-inflammatory properties and prevent colitis in a TNBS model. We compared the immunomodulatory properties of strain A2-165 to four different *F. prausnitzii* isolates and eight abundant intestinal commensals using human dendritic cells (DCs) and mouse BMDCs *in vitro*. Principal component analysis revealed that the cytokine response to *F. prausnitzii* A2-165 is distinct from the other strains in eliciting high amounts of IL-10 secretion. The mouse DNBS model of relapsing IBD was used to compare the protective effects of *F. prausnitzii* A2-165 and *Clostridium hathewayi,* a low secretor of IL-10, on the Th1-driven inflammatory response to DNBS; attenuation of disease parameters was only observed with *F. prausnitzii.* In an *in vivo* mouse model of nasal tolerance to ovalbumin, *F. prausnitzii* A2-165 enhanced ovalbumin-specific T cell proliferation and reduced the proportion of IFN-γ^+^ T cells in CLNs. Similarly, *in vitro F. prausnitzii* A2-165 stimulated BMDCs increased ovalbumin-specific T cell proliferation and reduced the number of IFN-γ^+^ T cells. These mechanisms may contribute to the anti-inflammatory effects of *F. prausnitzii* in colitis and support the notion that this abundant bacterium might contribute to immune homeostasis in the intestine via its anti-inflammatory properties.

The human gastrointestinal tract is colonized by several hundred different species of commensal bacteria, referred to as the microbiota, which reaches densities of 10^12^ bacteria per gram of luminal content in the colon^1^,^2^. Major changes in the microbiota are associated with a number of intestinal-related disorders including inflammatory bowel disease (IBD), irritable bowel syndrome, autoimmune disease, diabetes and colorectal cancer^3^.

In the healthy intestine, commensal bacteria are predominantly compartmentalized to the lumen, but can interact with immune cells associated with the epithelium or in mucosa-associated lymphoid tissues such as Peyer’s patches (PPs) or gut-draining mesenteric lymph nodes (MLNs). At these sites, antigen-presenting cells (APCs), such as dendritic cells (DCs), can sample microbes and food proteins and prime mucosal naive T cells driving their activation, clonal expansion and differentiation. In steady-state conditions, regulatory T cell (Treg) conversion in response to the presentation of harmless protein antigens occurs with a higher frequency in PPs and MLNs when compared to non-mucosa-draining lymphoid tissue. It has long been known that this preferential tolerance induction at mucosal sites is dependent on microbial sensing^4^,^5^.

Over the past years, it has become apparent that not all components of the microbiota are equal in terms of their impact on host physiology. For example, colonization of germ-free mice with the mouse commensal segmented filamentous bacteria induces an increase in the number of CD4^+^ T cells, in particular T helper 1 cells (Th1), Th17 and Foxp3^+^ Tregs in the small intestine and colon^6^. Conversely, colonization of germ-free mice with 17 Clostridia isolated from the human microbiota induced Tregs expansion and differentiation by inducing an increase in TGF-β^7^. Accumulation of colonic IL-10 secreting, Foxp3^+^ Tregs is also induced by colonization of germ-free mice with *Bacteroides fragilis*^8^. This implies that commensal bacteria may act upon mucosal T cells at different stages during their activation, differentiation as well as accumulation and maintenance in the lamina propria (LP).

The microbiota of patients with active Crohn’s disease (CD) and ulcerative colitis (UC), both forms of IBD, have increased abundance of Proteobacteria and lower faecal counts of Firmicutes compared to healthy individuals^9^. The Proteobacteria phylum includes pathobionts of *Escherichia coli* that contribute to the pathophysiology of the disease^10^. Among the Firmicutes, *Faecalibacterium prausnitzii* (*Fp*), one of the most abundant intestinal bacteria in humans^11^,^12^,^13^, is reduced in CD and UC patients^14^,^15^. *Fp* strain A2-165 has been shown to attenuate colitis induced by 2,4,6-trinitrobenzenesulfonic acid (TNBS) or dinitrobenzenesulfonic acid (DNBS) in mice and to induce a relatively high IL-10 to IL-12p70 cytokine ratio in human peripheral blood mononuclear cells (hPBMCs)^14^,^16^. *Fp* along with certain members of the Clostridium cluster XIVa are prominent butyrate producers in the intestine^17^. Butyrate is a primary energy source for the epithelial cells lining the colon^18^ and appears to have both anti-inflammatory and cancer chemopreventive activities^17^,^18^,^19^.

DCs and T cells are instrumental in regulating tolerance and immunity in the intestinal mucosa but the effects of *Fp* on these cells have not yet been described. It is not known if the *Fp* strain A2-165 tested in the mouse colitis models is characteristic of other *Fp* strains or if responses differ to other members of Clostridium cluster IV and cluster XIVa isolated from the human colon. Furthermore, the mechanisms of the protective and anti-inflammatory effects of *Fp* have not yet been fully unravelled.

To address these questions, we have investigated and compared the immunomodulatory properties of *Fp* with other commensal bacteria on DCs and T cells *in vitro.* The immunomodulatory and TLR signalling properties of 13 commensal strains of the Firmicutes and Actinobacteria phyla, including five isolates of *Fp* were first evaluated using human monocyte-derived DCs (hDCs) and TLR expressing cell lines. Further immune response studies were then performed on a smaller number of strains using mouse bone marrow-derived DCs (BMDCs). Subsequently, *Fp* strain A2-165 and another butyrate producing Clostridium, *Clostridium hathewayi (Ch)* 82-B were tested *in vivo* for their anti-inflammatory effects in the DNBS model of chronic relapsing colitis and on mucosal T cell differentiation using a well characterized model for nasal tolerance induction to the antigen ovalbumin (OVA).

## Results

### Colonic bacteria of Clostridium clusters IV and XIVa induce differential cytokine responses in hDCs

Incubation of hDCs with the commensal bacteria (Table 1) induced hDC maturation and activation and increased surface expression of the markers CD83 and CD86 when compared with the control immature non-stimulated hDCs (Supplementary Fig. S1). Incubation with the bacteria did not significantly alter hDC viability compared to the unstimulated control cells (60–80% viability, not shown).

**Figure 1:**
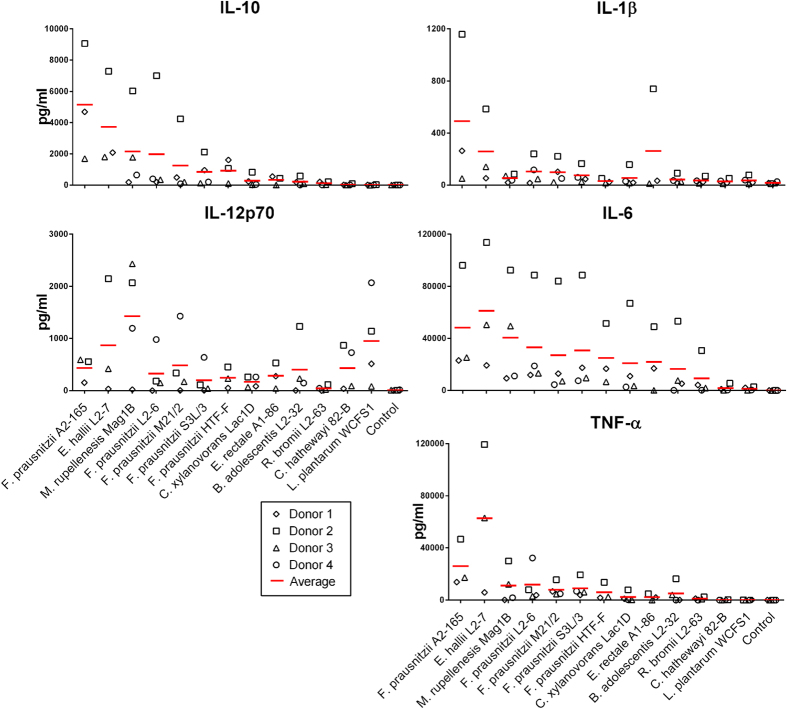
Cytokine secretion by hDCs. IL-10, IL-12p70, TNF-α, IL-1β and IL-6 were measured in the supernatant of hDCs from 3 to 4 donors after 48 h of incubation with the bacteria (bacterium to DC ratio 10:1, 10^6^ DCs/well in 24w plate). Each donor is represented with a different symbol and the red lines indicate the average of the donors.

Cytokines elicited by these strains result from the combination of TLR and NLR recognition of bacterial microbial associated molecular patterns (MAMPs) on the cell membrane as well as intra-cellularly after phagocytosis. Thus immune activation of hDCs was assessed by measuring the levels of IL-10, IL-12p70, IL-6, IL-1β and TNF-α secreted in the culture supernatant after incubation of the bacteria with hDCs from three to four different donors (Fig. 1). The largest amounts of IL-10 were induced by the *Fp* strains, *Eubacterium hallii* (*Eh*) L2-7 and *Megamonas rupellenesis* (*Mr*) Mag1B. In addition, these strains induced the largest amounts of TNF-α and IL-6. The *Fp* strains, *Eh* L2-7 and *Eubacterium rectale* (*Er*) A1-86 also induced relatively large amounts of IL-1β. *Lactobacillus plantarum* (*Lp*) WCFS1, *Mr* Mag1B and *Eh* L2-7 were among the most potent inducers of IL-12p70, whereas the *Fp* strains were among the poorer inducers (Fig. 1). Surprisingly, *Ruminococcus bromii* (*Rb*) L2-63, *Ch* 82-B and *Clostridium xylanovorans* (*Cx*) Lac1D induced small or undetectable amounts of some cytokines (Fig. 1) despite having effectively induced hDCs maturation (Supplementary Fig. S1). The five *Fp* strains had very different cytokines profiles. The highest IL-10 producer was strain A2-165, strains L2-6 and M21/2 induced high amounts of IL-10 but also IL-12p70 while strains S3L/3 and HTF-F induced intermediate to low amounts of both cytokines. The amounts of IL-10 induced by *Fp* strain A2-165 was 4.7 ng/ml in Donor 1, 1. 9 ng/ml in Donor 2 and 1.7 ng/ml in Donor 3. These amounts are relatively high compared to similar studies with probiotics and DCs or PBMCs^20^,^21^.

Bacteria and hDCs were incubated in the presence of penicillin and streptomycin to prevent contaminations and bacterial overgrowth so no short-chain fatty acids or other metabolites would be produced. However, we independently tested whether the *Fp* A2-165 cell free culture supernatants, bacterial medium or butyrate at concentrations similar to that measured in the bacterial culture supernatant would activate hDCs. In an initial experiment, the bacterial supernatants were found to be toxic for the hDCs at a concentration of 10% v/v (data not shown). This toxicity can be attributed to the high concentration of butyrate produced during bacterial fermentation^22^. *Fp* A2-165 culture supernatant (1.25 and 2.5% v/v) and butyrate (25 to 500 μM) did not affect cell viability or cytokine secretion by hDCs compared to non-stimulated cells (data not shown).

### Differential activation of TLRs by commensal bacteria

To investigate the potential relationship between cytokine profiles in hDCs and TLR signalling, bacteria were incubated with HEK293 reporter cell lines expressing different human TLRs and a reporter plasmid harbouring the luciferase gene under the control of the NF-κB promoter.

**Figure 2:**
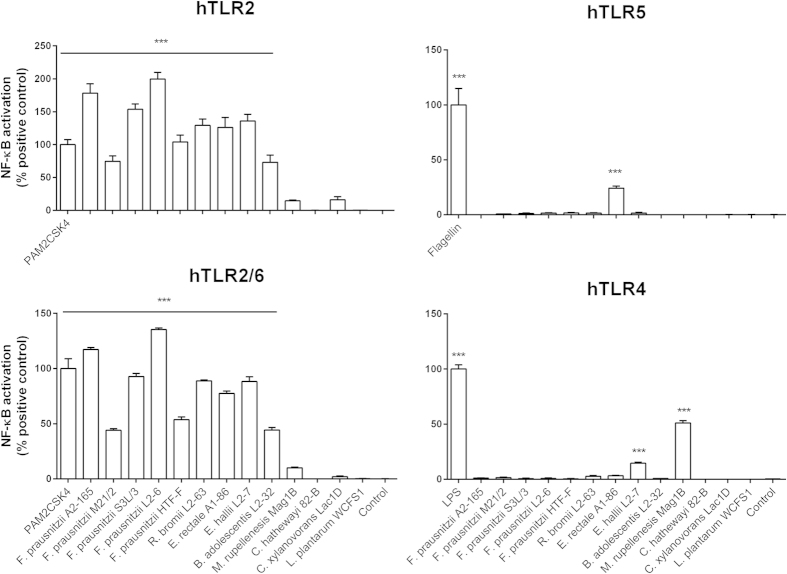
TLR signalling properties of the commensal bacteria. NF-κB activation was measured using a luminescence reporter in HEK293 cell lines expressing TLR2, TLR2/6, TLR5 and TLR4 after incubation with the bacteria (bacterium: cell ratio, 10:1). NF-κB activation is expressed as percentage of the positive control. Error bars represent SEM, n = 6, *indicates p < 0.05, **p < 0.01, ***p < 0.001 compared to the control.

The *Fp* strains, *Rb* L2-63, *Er* A1-86, *Eh* L2-7 and *Bifidobacterium adolescentis* (*Ba*) L2-32 induced high NF-κB activation in the TLR2/6 and TLR2 reporter cell lines (Fig. 2). Agonists of TLR2 include acylated lipoprotein anchors and potentially lipoteichoic acid present in Gram-positive and Gram-negative bacterial envelopes. As previously reported^23^, the flagellated strain *Er* A1-86^24^ induced NF-κB activation via TLR5 signalling. Surprisingly, *Eh* L2-7 and *Mr* Mag1B activated TLR4 signalling although they are considered Gram-positive. *Mr* Mag1B however, has been described previously as Gram-negative to Gram-variable^25^. *Ch* 82-B and *Cx* Lac1D did not induce NF-κB activation in any of the TLR assays. The relative capacity of the strains to elicit NF-κB activation through TLR signalling pathways correlated with the induction of cytokines in hDCs.

### Principal component analysis

**Figure 3:**
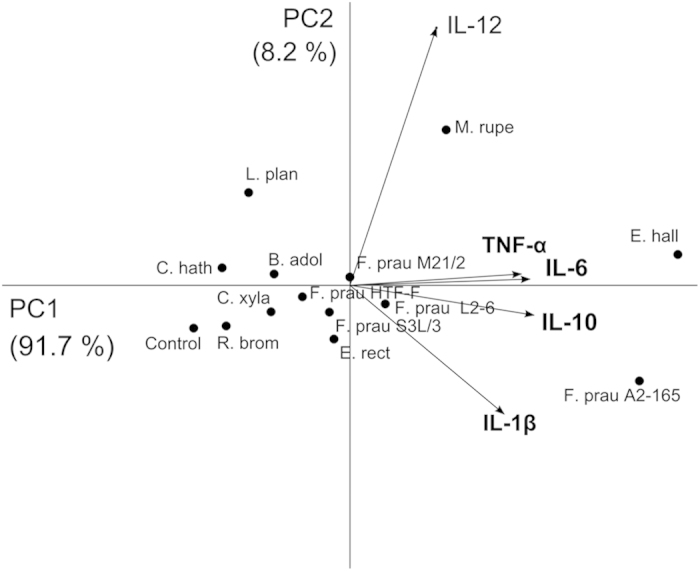
Principal component analysis (PCA) of the cytokine profiles induced by the commensal bacteria in hDCs. Each dot represents the average of cytokine levels of a commensal strain in hDCs. The variables (level of cytokines) are represented by vectors. The direction and length of a vector indicates how the variable contributes to the two principal components in the plot. (F. prau, *F. prausnitzii*; R. brom, *R. bromii* L2-63; E. rect, *E. rectale* A1-86; E. hall, *E. halli* L2-7; M. rupe, *M. rupellensis* Mag1B; C. hath, *C. hathewayi* 82-B; C. xyla, *C. xylanovorans* Lac1D; L. plan, *L. plantarum* WCFS1).

Principal component analysis (PCA) was performed to describe the variance of the cytokine levels (IL-10, IL-12p70, IL-6, IL-1β and TNF-α) induced by the bacteria after incubation with hDCs. The PC 1 and PC 2 of the PCA describe 99.9% of the variance between the cytokine levels induced by the bacteria. The coefficients of PC 1 revealed that the contribution of the different cytokines to the variance was almost equal, the coefficient associated with IL-12p70 was 0.2 while for the other cytokines the coefficient was in average 0.48. The PC 1, PC 2 score plot (Fig. 3) revealed that the majority of the bacteria are grouped in the centre of the plot, while *Fp* A2-165, *Eh* L2-7, *Mr* Mag1B and *Lp* WCFS1 are clearly separated from the other bacteria and from each other reflecting their capacity to induce distinct cytokine profiles in hDCs. Furthermore, PCA revealed that the immune response induced by *Fp* A2-165 is very different from the other *Fp* strains tested.

### Mouse BMDC cytokine responses to commensal bacteria

In order to select a strain to compare with *Fp* A2-165 in subsequent *in vivo* experiments, the immune response of mouse BMDCs incubated with *Fp* A2-165 and five other human commensal bacterial strains was measured. The candidate strains were chosen on the basis of cytokines produced in culture with hDCs. *Fp* A2-165 was the highest inducer of IL-10 release by hDCs, *Ch* 82-B and *Cx* Lac1D induced low or undetectable cytokine responses, *Lp* WCFS1 was one of the highest inducers of IL-12p70 release and *Er* A1-86 and *Ba* L2-32 induced intermediate levels of cytokines compared to the other strains (Fig. 1).

**Figure 4:**
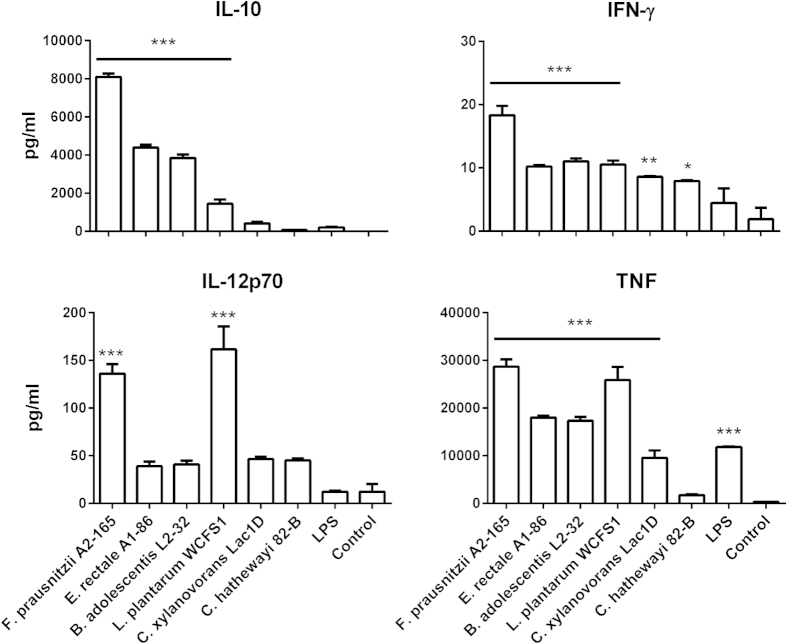
Cytokine secretion by mouse BMDCs. IL-10, IL-12p70, TNF and IFN-γ were measured in the supernatant of BMDCs (5 × 10^5^ BMDCs/well in 24w plate) after 24 h of incubation with bacteria (bacterium: BMDC, 10:1). Error bars represent SEM, n = 3, ***indicates p < 0.001, **p < 0.01, *p < 0.05 compared to the control.

All the strains induced increased surface expression of the co-stimulatory molecules CD86 and CD40 by BMDCs compared to untreated BMDCs (Supplementary Fig. S2). Culture of BMDCs with *Fp* A2-165 induced the largest amount of IL-10 release, followed by *Er* A1-86 and *Ba* L2-32. IL-12p70 release by BMDCs was mostly induced by *Fp* A2-165 and *Lp* WCFS1. However, for all the strains tested the levels of IL-12p70 were around 10 fold lower than those obtained using hDCs (Fig. 4). The amounts of IFN-γ in the culture supernatants of stimulated BMDCs were low but high levels of TNF release were induced by virtually all bacterial strains with *Lp* WCFS1 and *Fp* A2-165 being the strongest inducers while *Ch* 82-B and *Cx* Lac1D were low to weak inducers. *Ch* 82-B was selected for comparison to *Fp* A2-165 in subsequent experiments *in vitro* and *in vivo* as it is capable of activating immune cells but a weak inducer of IL-10 and other cytokines.

### *F. prausnitzii* A2-165 but not *C. hathewayi* 82-B attenuates inflammation in a chronic relapsing model of colitis

**Figure 5:**
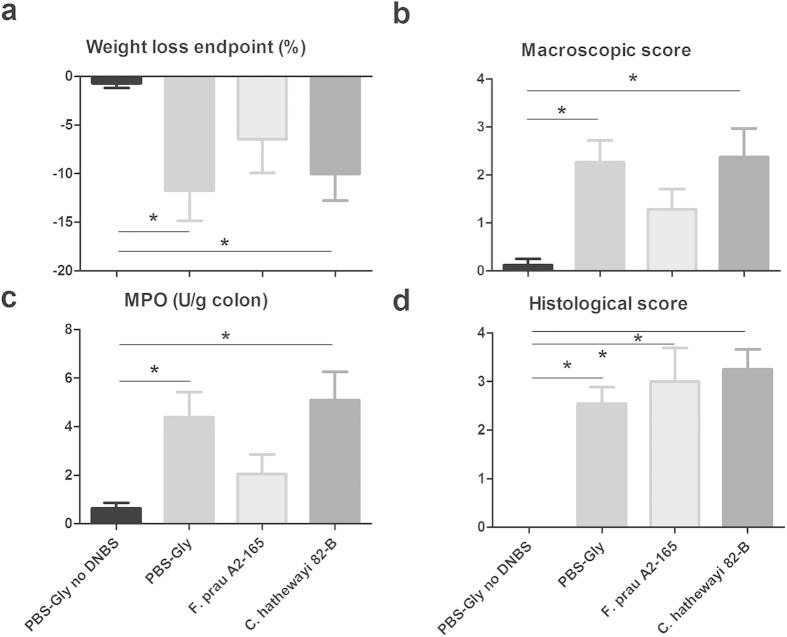
Effects of *F. prausnitzii* A2-165 and *C. hathewayi* 82-B on DNBS induced colitis. Percentage of weight loss at the end of the experiment (**a**), macroscopic scores (**b**), MPO activity (**c**) and histological scores (**d**) in control non-inflamed (PBS-Gly no DNBS), control inflamed (PBS-Gly), *F. prausnitzii* A2-165, *C. hathewayi* 82-B treated mice. Error bars represent SEM, n = 8, *indicates p < 0.05 compared to the control PBS-DNBS.

The capacity of *Fp* A2-165 to attenuate parameters of inflammatory colitis was investigated using a recently described model of chronic relapsing colitis^16^ and compared to the one of *Ch* 82-B. *Ch* 82-B was selected as it also produces butyrate but does not induce significant levels of IL-10 in BMDCs, or hDCs compared to *Fp* A2-165. Mice were administered a first dose of DNBS, and, after full recovery (day 10), were administered either *Fp* A2-165, *Ch* 82-B or ethanol intragastrically for other 10 days. On day 20 mice received a second lower dose of DNBS to induce a Th1-driven inflammatory response to the hapten. The weight loss of the *Fp* A2-165 treated mice was significantly smaller than the one of the DNBS control mice at the endpoint of the experiment (Fig. 5a). Additionally, the *Fp* A2-165 treated mice gained body weight significantly more rapidly than *Ch* 82B treated or DNBS control mice after the second administration of DNBS (Supplementary Fig. S4). In the *Fp* A2-165 treated mice, the macroscopic disease scores were significantly lower than in the DNBS control and in the *Ch* 82-B groups (Fig. 5b). Additionally, myeloperoxidase (MPO), an enzyme produced mainly by neutrophils and used as a marker of neutrophilic infiltration, was significantly lower in the *Fp* A2-165 treated group than in the other groups, suggesting reduced inflammatory responses leading to the recruitment of neutrophils (Fig. 5c). *Fp* A2-165 and *Ch* 82-B had no effect on histological damages (Fig. 5d).

### *In vivo* CD4^+^ T cell responses

To investigate whether the attenuation of certain disease scores in the DNBS model was due to the modulation of the mucosal T cell response by *Fp* A2-165, we used an *in vivo* model of nasal tolerance induction^26^. Nasal tolerance is known to have many similarities to oral tolerance induction^26^,^27^ and application of bacteria via the nasal route effectively targets responses in the nose-draining CLNs. In short, BALB/c acceptor mice were adoptively transferred with CFSE labelled naive DO11.10 OVA T cell receptor (TCR) transgenic T cells and after 24 h, received an intranasal (i.n.) application of either the bacteria together with OVA or OVA alone. After 72 h, single cell suspensions isolated from CLNs and spleens were analysed to assess the proliferation and phenotype of OVA-specific T cells (OVA-T cells).

**Figure 6:**
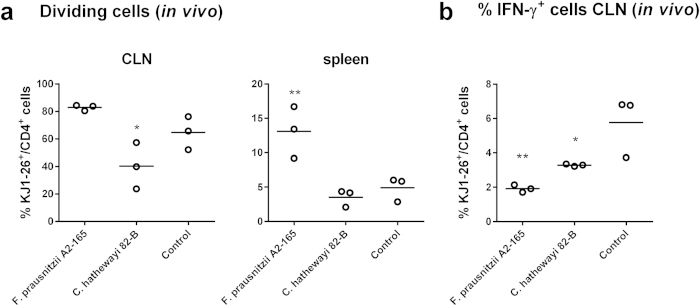
Percentage of dividing and IFN-γ^+^ OVA-T cells *in vivo*. CFSE labelled naive OVA-T cells (KJ1-26^+^/CD4^+^) were adoptively transferred in BALB/c mice, after 24 h, mice were administered i.n. with bacteria plus OVA and after additional 72 h, OVA-T cells were isolated from cervical lymph nodes (CLNs) and spleens and analysed. (**a**) Percentage of dividing OVA-T cells (KJ1-26^+^/CD4^+^) isolated from CLNs or spleens. (**b**) Percentage of IFN-γ^+^ OVA-T cells (KJ1-26^+^/CD4^+^) isolated from CLNs. *indicates p < 0.05, **indicates p < 0.01 compared to the control administered OVA alone.

In *Fp* A2-165 treated mice, the percentage of dividing OVA-T cells in the CLNs was increased compared to the control mice administered OVA alone. In the spleen of control mice, that received OVA i.n., a population of OVA-T cells was found in the third, fourth or fifth division. In agreement with previous observations, no CFSE fluorescence was detected corresponding with T cells in first or second division. Therefore, this population reflects cells that have differentiated in the CLNs and entered the circulation upon exiting the CLNs. Administration of *Fp* A2-165 together with OVA significantly increased the number of divided OVA-T cells isolated from spleens compared to control mice (Fig. 6a and Supplementary Fig. S5). Together, these data demonstrate that i.n. administration of *Fp* A2-165 enhances the number of OVA-T cells that divide in the CLNs. In contrast, *Ch* 82-B administration significantly decreased the proliferation of OVA-T cells isolated from CLNs compared to the control and *Fp* A2-165 (Fig. 6a). This effect was also observed in a second independent experiment although the differences were not statistically significant (Supplementary Fig. S3). Increased numbers of divided OVA-T cells were not detected in the spleen of mice administered *Ch* 82-B (Fig. 6a).

I.n. administration of either *Fp* A2-165 or *Ch* 82-B together with OVA significantly decreased the number of IFN-γ secreting OVA-T cells isolated from the CLNs. In particular, *Fp* A2-165 induced an approximately three-fold reduction in the percentage of IFN-γ secreting cells (Fig. 6b). However, these bacteria did not affect the intracellular expression of Foxp3, IL-17, or IL-10 in OVA-T cells isolated from the CLNs (not shown).

**Figure 7:**
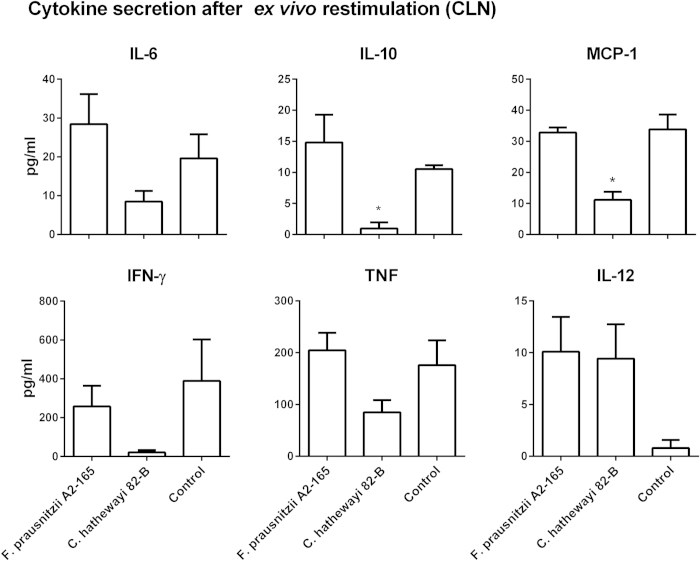
Cytokine secretion by OVA-T cells after *ex vivo* re-stimulation. CFSE labelled naive OVA-T cells were adoptively transferred in BALB/c mice, after 24 h, mice were administered i.n. with bacteria plus OVA and after additional 72 h, OVA-T cells were isolated from CLNs and re-stimulated with OVA for additional 24 h and then analysed. *indicates p < 0.05 compared to the control administered OVA alone.

Overall re-stimulation of CLN cells led to low and variable levels of cytokine release. This is likely to be due to the low frequency of OVA-T cells within the total CLN cell population. Notably, CLN cells of *Ch* 82-B treated mice released lower amounts of cytokines but this was only significant for IL-10 and MCP-1 (Fig. 7).

### Effects of *F. prausnitzii* A2-165 and *C. hathewayi* 82-B on T cell differentiation in BMDC-T cell co-cultures

In order to investigate whether the effects of *Fp* A2-165 and *Ch* 82-B on activation, differentiation and modulation of T cell responses *in vivo* could be observed in an *in vitro* assay we cultured BMDCs in the presence or absence of the bacteria or LPS for 24 h and then OVA was added for a further 6 h. Finally, naive CFSE labelled CD4^+^ OVA-T cells were added for an additional 72 h.

**Figure 8:**
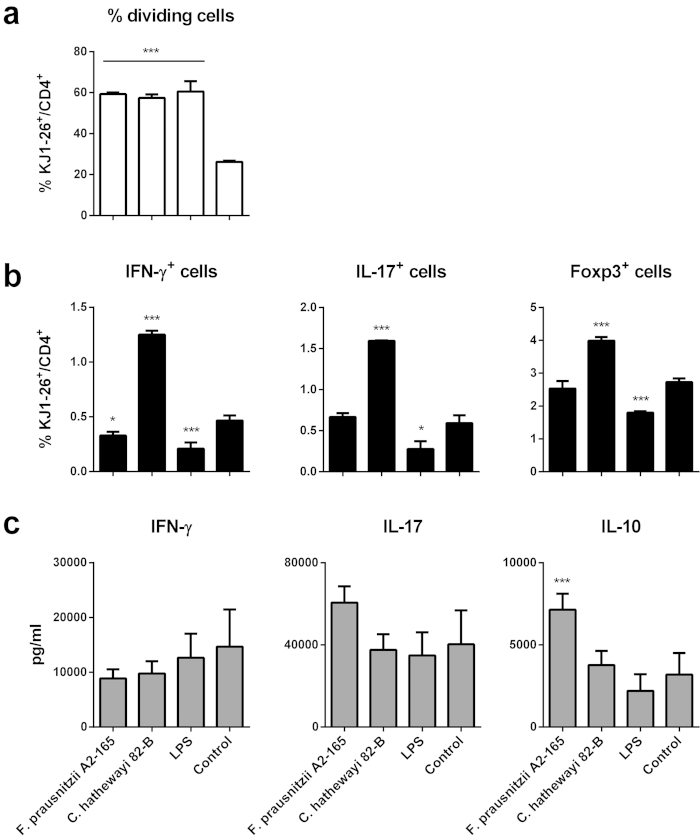
OVA-T cells (KJ1-26^+^/CD4^+^) division (a), IFN-γ^+^, IL-17^+^, Foxp3^+^ cells (b) and cytokine secretion (c) after incubation with BMDCs pre-stimulated with bacteria (bacterium: BMDC, 10: 1). BMDCs (5 × 10^4^ BMDCs/well in 96w plate) were loaded with OVA (0.5 mg/ml) and cultured in the presence or absence of the bacteria or LPS and after 24 h, CFSE labelled OVA-T cells (5 × 10^5^ T cells/well) were added, after 72 h cytokine secretion in the supernatant and intracellular markers were measured. Error bars represent SEM, n = 3, *** indicates p < 0.001 compared to the control, *p < 0.05 compared to the control. Intracellular IL-10 staining was negative for all samples.

All conditions of BMDC stimulation increased the number of OVA-T cells undergoing proliferation compared to the unstimulated BMDC control. No significant differences in the percentage of dividing T cells were observed between BMDC stimulation with LPS, *Fp* A2-165 or *Ch* 82-B (Fig. 8a and Supplementary Fig. S6).

As observed *in vivo* the intracellular staining of OVA-T cells revealed that *Fp* A2-165 decreased the percentage of total IFN-γ^+^ T cells compared to the control but had no effect on IL-17^+^ and Foxp3^+^ T cells (Fig. 8b). Notably, BMDCs pre-incubated with *Fp* A2-165 induced increased IL-10 secretion during OVA-T cell proliferation (Fig. 8c) compared to the control. However, no significant changes were observed in the amounts of IFN-γ and IL-17 in any of the culture supernatants compared to the control (Fig. 8c). None of the bacteria altered the levels of secreted IL-2, IL-6 or TNF compared to the control (not shown). In contrast to *Fp*, *Ch* 82-B induced an increase in the percentage of IFN-γ^+^, IL-17^+^ and Foxp3^+^ T cells compared to the control (Fig. 8b) which is not consistent with results with this strain *in vivo*. It may be that the *in vitro* immune assays are not predictive for the complex situation *in vivo* where stromal cytokines and factors also influence APC phenotypes. Another possibility is that this inconsistency is due to the fact that *Ch* 82-B possesses almost no TLR signalling activity (Fig. 2) but can strongly activate hDCs and BMDCs *in vitro* (Supplementary Fig. S1 and S2) resulting in no or low amounts of cytokine secretion. Thus activation of hDCs and BMDCs by *Ch* 82-B is presumably the consequence of phagocytosis and intracellular NLR activation.

## Discussion

Members of the Firmicutes, including members of the Clostridium clusters IV and XIVa, are reduced in abundance in the fecal microbiota of IBD patients^28^,^29^. Additionally, mucosal abundance of *Fp*, a member of Clostridium cluster IV, has been correlated with increased recurrence of colitis in CD patients^14^. *Fp* strain A2-165 and its culture supernatant were previously shown to attenuate colitis in mouse models. The incubation of hPBMCs with *Fp* A2-165 induced a relatively high IL-10 to IL-12p70 cytokine ratio, suggesting that the bacterium might have an effect on DCs and induction of T cells in the mucosal lymphoid tissue^14^,^16^. Furthermore, IL-10 produced by APCs in the LP has been reported to enhance the suppressive activity of Foxp3^+^ Tregs in the mucosa^30^.

We investigated the immunomodulatory properties of five *Fp* strains and eight other abundant intestinal commensal bacteria of Clostridium clusters IV and XIVa. Other Clostridia, when administered as a spore preparation have been shown to induce the expansion and activation of Tregs after colonization of germ-free mice^31^. All the bacteria tested were able to induce efficient maturation and activation of hDCs (Supplementary Fig. S1). In hDCs, the highest levels of IL-10 were induced by *Fp* A2-165, *Eh* L2-7 and *Mr* Mag1B. *Fp* A2-165 was among the weakest inducers of IL-12p70 while *Mr* Mag1B and *Eh* L2-7 were among the strongest inducers. Additionally, these strains induced the largest amounts of TNF-α and IL-6. The *Fp* strains, *Eh* L2-7 and *Er* A1-86 induced also relatively large amounts of IL-1β (Fig. 1). PCA revealed that *Fp* A2-165, *Mr* Mag1B and *Eh* L2-7 cause distinct effects on the immune cells when compared to the other strains. These differential effects were due to the high levels of IL-10 induced by *Fp* A2-165, the high levels of IL-12p70 and TNF-α induced by *Mr* Mag1B and the high levels of IL-10 and IL-12p70 induced by *Eh* L2-7. Furthermore, *Fp* A2-165 is separated from the other *Fp* strains (Fig. 3).

In general, high TLR signalling activity was associated with the ability of commensal bacteria to induce cytokines in the DC assays. TLR4 signalling was induced by *Eh* L2-7 and *Mr* Mag1B, belonging to the Firmicutes lineage of bacteria, which are generally considered as Gram-positive. However, due to their unique cell wall composition, upon Gram staining these bacteria appeared to be Gram-negative to Gram-variable^24^,^25^. *Ch* 82-B and *Cx* Lac1D induced low NF-κB activation in the TLR assays and low or undetectable cytokines in the DC assay (Figs 1 and 2) despite the fact that they induced activation and maturation of DCs (Supplementary Fig. S1). These strains might express MAMPs that are shielded or not recognized by the TLRs tested or might possess immunomodulatory components that inhibit activation of the NF-κB pathway. This appears to be a unique property and may be an evolutionary adaptation to avoid immune recognition by the host as proposed for *Bacteroides fragilis*^32^.

*Fp* A2-165 has been previously reported to be anti-inflammatory and attenuate inflammation in mouse models of colitis^14^,^16^, therefore we compared its protective effects in the DNBS model of chronic relapsing colitis with that of *Ch* 82-B, a butyrate producing organism that induces only low amounts of IL-10 secretion in hDCs and BMDCs. In the *Fp* A2-165 treated mice, body weight loss, tissue MPO and macroscopic disease scores were attenuated compared to the control and *Ch* 82-B groups (Fig. 5). To investigate whether the attenuation of inflammation parameters in the DNBS model was due to modulation of the mucosal T cell response by *Fp* A2-165, we i.n. inoculated OVA or OVA together with the bacteria into BALB/c mice adoptively transferred with naive OVA-TCR transgenic T cells. After 72 h, single cell suspensions isolated from CLNs and spleens were analysed to assess the proliferation and phenotype of OVA-T cells. *Fp* A2-165 enhanced the number of OVA-T cells that divide in the CLNs and decreased the percentage of IFN-γ secreting T cells (Fig. 6). The effects of *Fp* on T cell differentiation *in vivo* may be due to the uptake of bacteria or microbial factors (including MAMPs) resulting in increased DC activation as seen in the *in vitro* experiments (Supplementary Fig. S1 and S2). The activated DCs may then migrate from PPs or LP to drive enhanced antigen presentation of OVA in the CLNs. Although *Fp* A2-165 administration increased the percentage of dividing T cells *in vivo*, it decreased the percentage of IFN-γ^+^ T cells without affecting Foxp3^+^ or IL-17^+^ T cells (Fig. 6). This finding suggests that *Fp* A2-165 modulated the phenotype of mucosal DCs that migrate from the LP to suppress IFN-γ differentiation in the draining CLNs. Unfortunately we were not able to detect intracellular IL-10 staining in CLNs cells. IL-10 may suppress differentiation of IFN-γ secreting cells in the CLNs once the cells have proliferated and re-express their IL-10R. Future experiments using *in vivo* blocking with anti-IL-10R antibodies would provide an answer to this hypothesis.

Strikingly, the effects of *Fp* A2-165 on T cell proliferation and differentiation of IFN-γ secreting T cells *in vivo* were also observed *in vitro* using *Fp* A2-165 stimulated BMDCs co-cultured with naive D011.10 T cells. In these co-cultures, secretion of IL-10 was significantly higher than the control when the BMDCs were pre-stimulated with *Fp* A2-165 but not with other stimuli (Fig. 8). The concomitant enhanced proliferation and high levels of IL-10 secretion in BMDC-T cell co-cultures may seem counter intuitive as IL-10 is known to suppress T cell proliferation. However, the onset of T cell proliferation may not be sensitive to IL-10 suppression because the cells down regulate expression of the IL-10Rα chain^33^.

In contrast to *Fp* A2-165*, Ch* 82-B reduced the number of proliferating OVA-T cells in the CLNs *in vivo* (Fig. 6). This finding was in contrast to the *in vitro* results showing that *Ch* 82-B stimulated BMDCs were highly effective in stimulating T cell proliferation. Indeed, the percentage of T cells staining positive for intracellular markers of different T cell subsets (i.e. IFN-γ, IL-17 and Foxp3) were significantly higher for T cells incubated with BMDCs pre-stimulated with *Ch* 82-B than BMDCs pre-stimulated with *Fp* A2-165, LPS or left unstimulated (Fig. 8). The reasons for this are not clear but may be due to inhibitory effects of *Ch* on the migration of DCs from the mucosa to the draining lymph nodes, which is not mimicked *in vitro.*

In conclusion, we demonstrated that *Fp* A2-165 has a strong capacity to induce IL-10 in human and murine DCs and influence T cell differentiation *in vitro* and *in vivo*. The reduction in IFN-γ^+^ cells *in vivo* and the IL-10 induction in the LP cells of damaged intestinal tissue could be a mechanisms contributing to the suppressive effect on inflammation in mouse colitis models^14^,^16^. Moreover, these findings suggest that this normally abundant bacterium might contribute to immune homeostasis in the intestine via its anti-inflammatory properties.

## Materials and Methods

### Bacterial strains and culturing conditions

All the commensal bacteria used (Table 1) were isolated from human faeces and identified by 16S rRNA sequencing as previously described^2^. *L. plantarum* WCFS1 was isolated from human saliva. The commensal bacteria were maintained at 37 °C on YCFAG in an anaerobic tent. Bacteria were grown in YCFAG broth to an optical density at 600 nm (A600) of 0.8, which corresponds to the late exponential or early stationary growth phase. *L. plantarum* WCFS1 was cultured overnight to stationary phase in MRS broth (MRS, Merck, Darmstadt, Germany) at 37 °C. Bacteria were harvested by centrifugation, washed in PBS, resuspended in PBS containing 20% glycerol and stored at −80 °C prior to use in the assays. Bacteria were quantified by fluorescent *in situ* hybridization (FISH) or phase contrast microscopy. All buffers and media used for the anaerobic bacteria were deoxygenated by flushing with oxygen free nitrogen for 30 minutes.

### Human DCs assays

This study was approved by Wageningen University Ethical Committee and was conducted in accordance with the principles of the ‘Declaration of Helsinki’ (52nd WMA General Assembly, Edinburgh, Scotland, October 2000). Buffy coats from healthy blood donors were obtained from the Sanquin Blood bank in Nijmegen (The Netherlands). A written informed consent was obtained from each volunteer before sample collection. See Supplementary Methods.

### TLR signalling assays

See Supplementary Methods.

### Mice

For the DNBS induced colitis, animal experiments were approved by the Ethic Committee of Jouy en Josas research centre. Male C57BL/6 mice (6 weeks old) were purchased from Janvier Labs and maintained at the animal facility under specific pathogen-free conditions. Mice were housed under standard conditions for a minimum of 1 week before experimentation.

For the *in vivo* nasal tolerance and the *in vitro* BMDCs and T cells assays, animal experiments were approved by the Animal Experimental Committee of the Erasmus Medical Centre. BALB/c mice were obtained from Charles River Laboratories (Maastricht, The Netherlands) and kept in the Erasmus University Medical Centre. DO11.10 transgenic mice on BALB/c background, which have a T cell receptor (TCR) specific for the OVA 323-339 peptide, were bred at the Erasmus University Medical Centre. Mice were kept under specific pathogen-free housing conditions.

Animals were handled in accordance with the general principles and guidelines recommended by the European Community (Directive 86/609/EEC) and current French legislation (Law 87–848) or Dutch legislation (The Experiments on Animals Act, 1997).

### Mouse BMDCs assays

See Supplementary Methods.

### Induction of DNBS colitis and bacteria administration

The DNBS induced chronic colitis model was described by Martin *et al.*^16^. Briefly, mice were anesthetized and a 4 cm long catheter (Ecimed, France) was attached to a 1 ml-syringe and inserted 3.5 cm into the colon. Colitis was induced by intrarectal injection of 150 mg/kg of DNBS solution (Sigma) in 30% ethanol (EtOH). Control mice (that did not develop colitis) received only EtOH. Mice were supplied with 6% sucrose in the drinking water for the first 3 days after DNBS injection to prevent dehydration (DNBS period). Ten days following DNBS injection, 200 μL containing either 1 × 10^9^ bacteria or 200 μL of PBS were administered intragastrically daily for 10 days. Colitis was reactivated 21 days after the first DNBS injection (recovery period) with a second injection of 150 mg/kg of DNBS solution, control mice received only EtOH. Mice weight was recorded through all the experiment, each group consisted of 8 mice.

### Macroscopic scores, colonic histology and myeloperoxidase activity

Severity of colitis was determined as previously described^16^. See Supplementary Methods.

### Purification of DO11.10 T-cells

See Supplementary Methods.

### *In vitro* assays with DO11.10 T cells

See Supplementary Methods.

### Adoptive transfer of DO11.10 T cells and intranasal challenge

For the *in vivo* nasal tolerance study, acceptor BALB/c mice received 6 × 10^6^ CFSE labelled KJ1-26^+^ CD4^+^ T cells in 200 μl saline (NaCl 0.9%, Brown) by injection in the tail vein. DO11.10xRag-/- T cells were isolated as described above. Twenty-four h after the adoptive transfer, mice were administered i.n. with 2 × 10^8^ bacteria in 15 μl of saline containing 400 μg of OVA or 15 μl of saline with OVA as a control. Throughout the *in vivo* experiment, groups of mice that received different treatments were housed separately. After 72 h, mice were sacrificed, nose-draining superficial and deep cervical lymph nodes (CLNs) and spleens were collected and single cell suspensions were analysed by flow cytometry for cell division and stained for surface or intracellular protein detection or used for *ex vivo* T-cells restimulation.

### *Ex vivo* DO11.10 T-cell restimulation

See Supplementary Methods.

### Flow cytometry

See Supplementary Methods.

### Statistics and principal component analysis

Differences in NF-κB activation in the TLR assays, cytokine secretion, surface marker expression and percentage of dividing or positive cells were analysed comparing samples treated with the bacteria or LPS with the untreated control. Data were analysed by one way Anova followed by Dunnett’s multiple comparison test for the *in vitro* assays or Newman-Keuls multiple comparison test for the *in vivo* assays using the GraphPad PRISM (GraphPad Software). PCA was used to analyse the variance of the level of cytokines along the different types of bacteria studied. PCA was computed on the standardized data by using the function princomp of the software Matlab.

## Additional Information

**How to cite this article**: Rossi, O. *et al.* *Faecalibacterium prausnitzii* A2-165 has a high capacity to induce IL-10 in human and murine dendritic cells and modulates T cell responses. *Sci. Rep.* **6**, 18507; doi: 10.1038/srep18507 (2016).

## Supplementary Material

Supplementary Information

## Figures and Tables

**Table 1:** Phylum, family and phylogenetic cluster of the bacteria used.

Bacterial strain	Phylum	Family	Phylogenetic cluster	Ref.
*F. prausnitzii* A2-165	Firmicutes	Ruminococcaceae	Clostr. cluster IV	12
*F. prausnitzii* M21/2	Firmicutes	Ruminococcaceae	Clostr. cluster IV	34
*F. prausnitzii* S3L/3	Firmicutes	Ruminococcaceae	Clostr. cluster IV	34
*F. prausnitzii* L2-6	Firmicutes	Ruminococcaceae	Clostr. cluster IV	12
*F. prausnitzii* HTF-F	Firmicutes	Ruminococcaceae	Clostr. cluster IV	2
*R. bromii* L2-63	Firmicutes	Ruminococcaceae	Clostr. cluster IV	13
*E. rectale* A1-86	Firmicutes	Lachnospiraceae	Clostr. cluster XIVa	13
*E. hallii* L2-7	Firmicutes	Lachnospiraceae	Clostr. cluster XIVa	35
*B. adolescentis* L2-32	Actinobacteria	Bifidobacteriaceae		13
*Megamonas* sp. Mag1B 95,0% homology* with *M. rupellensis*	Firmicutes	Veillonellaceae		This work
*Clostridium* sp. 82B 95,4% homology* with *C. hathewayi*	Firmicutes	Clostridiaceae	Clostr. cluster XIVa	This work
*Clostridium* sp. Lac1D 90,5% homology* with *C. xylanovorans*	Firmicutes	Clostridiaceae	Clostr. cluster XIVa	This work
*L. plantarum* WCFS1	Firmicutes	Lactobacillaceae		

(Clostr, Clostridium); *based on the sequence homology of 850 nucleotides of the 16S rRNA.
