# Supplementary material for: Faecalibacterium prausnitzii A2-165 has a high capacity to induce IL-10 in human and murine dendritic cells and modulates T cell responses
Source: Sci Rep. 2016 Jan 4;6:18507. doi: 10.1038/srep18507 (PMC4698756; doi:10.1038/srep18507)
Supplement: Supplementary Information [file srep18507-s1.pdf]

Supplementary Information

Faecalibacterium prausnitzii A2-165 induces IL-10 in human and murine dendritic cells and modulate T cell responses

Oriana Rossi[1], Lisette A. van Berkel[2], Florian Chain[3,4], M. Tanweer Khan[5], Nico Taverne[1], Harry Sokol[3,4,6,7], Sylvia H. Duncan[8], Harry J. Flint[8], Hermie J. M. Harmsen[5], Philippe Langella[3,4], Janneke N. Samsom[2#] and Jerry M. Wells[1#*]

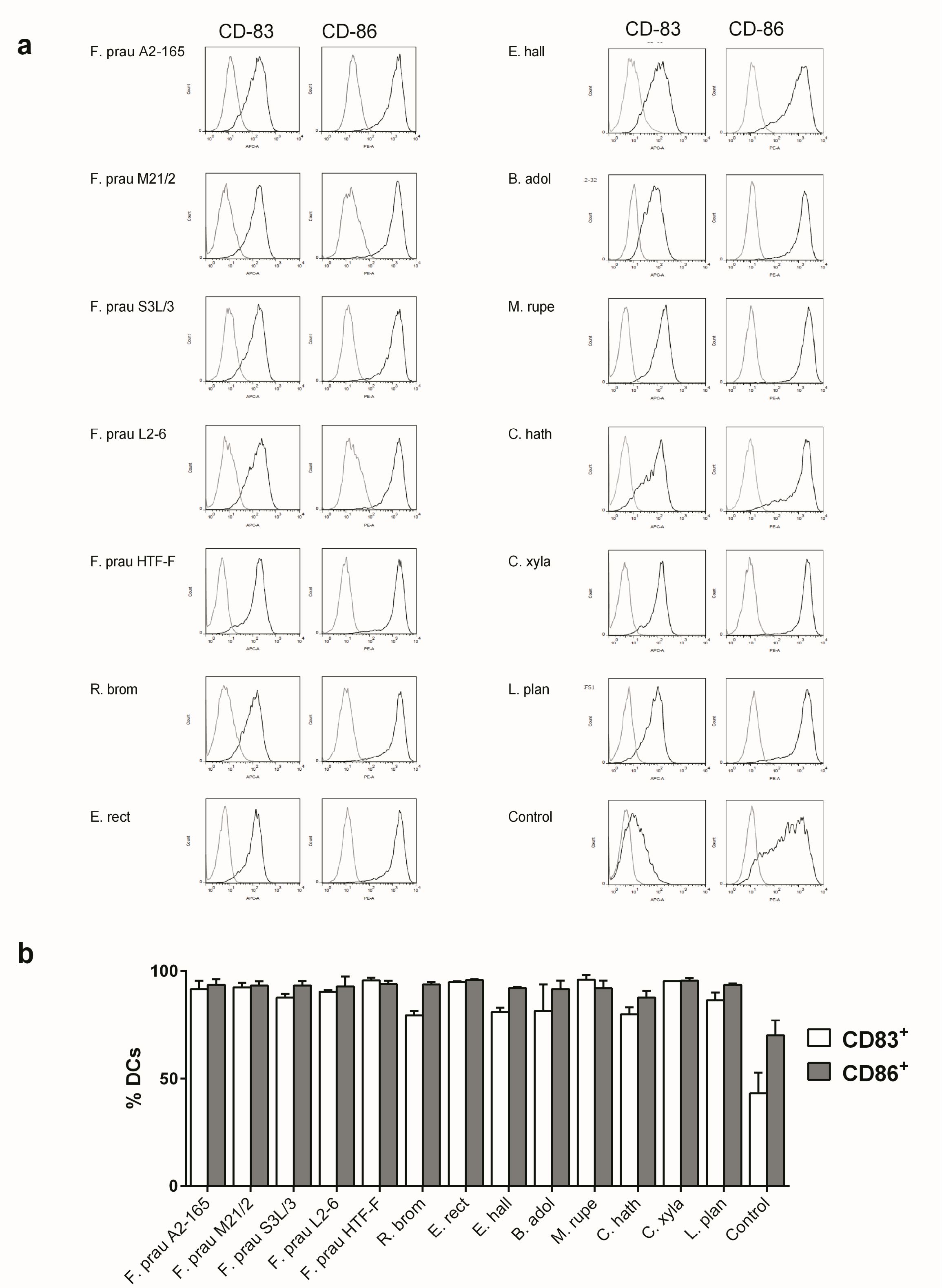

Supplementary Figure S1: CD83 and CD86 surface expression in hDCs. a) Representative histograms showing the expression of the surface markers CD83 and CD86 on hDCs. Grey lines represent the isotype controls and black lines the staining. b) Percentage of $CD83^+$ (in white) and $CD86^+$ (in grey) hDCs from 3 donors. Error bars represent SEM.

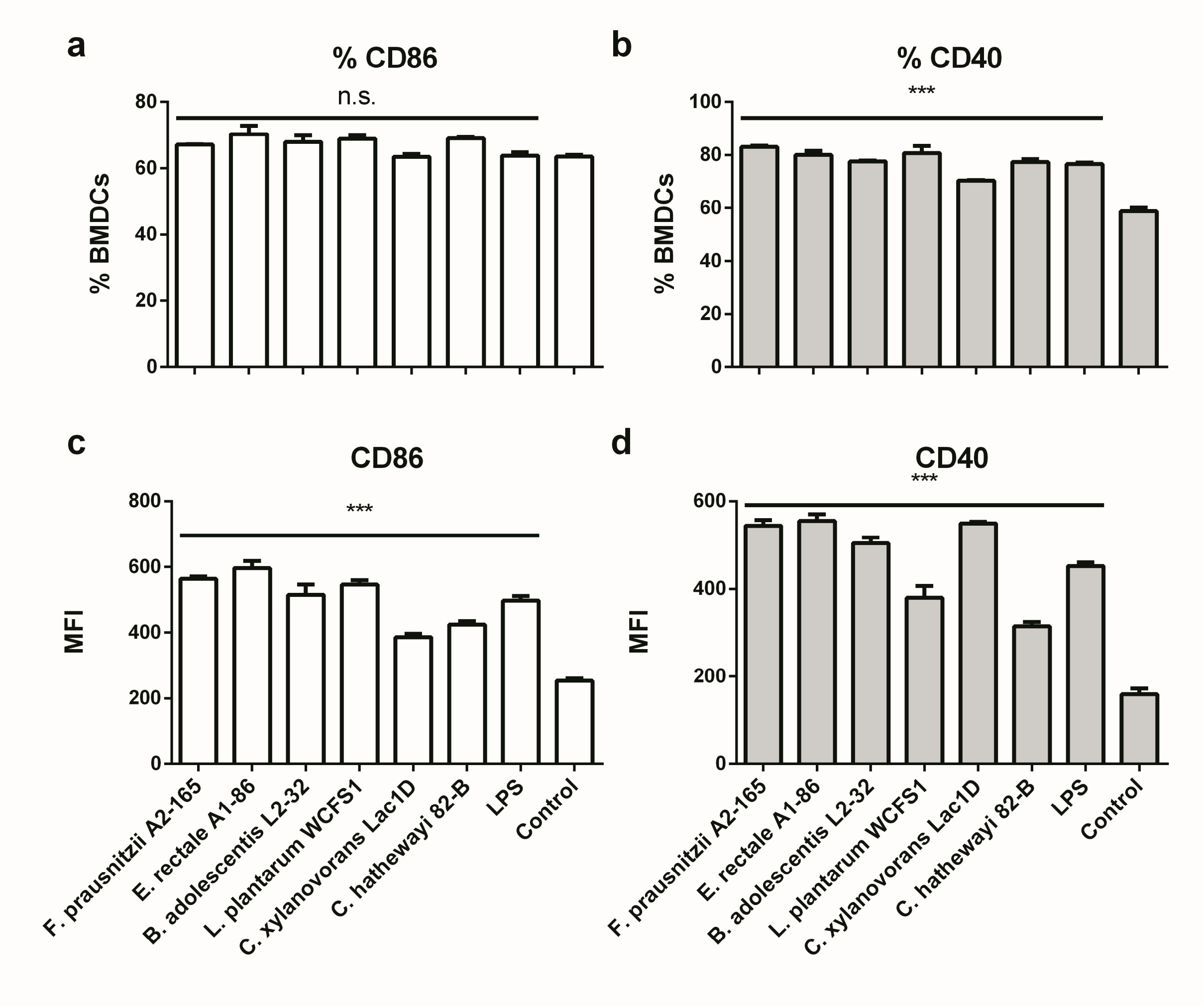

Supplementary Figure S2: CD86 and CD40 surface expression in BMDCs. Percentage of $CD86^+$ (a) or $CD40^+$ (b) BMDCs and mean fluorescence intensity (MFI) of CD86 (c) and CD40 (d) after 24 h of incubation with bacteria (bacterium: BMDC, 10:1). Error bars represent SEM, n=3, *** indicates p<0.001, n.s. indicates non-significant compared to the control.

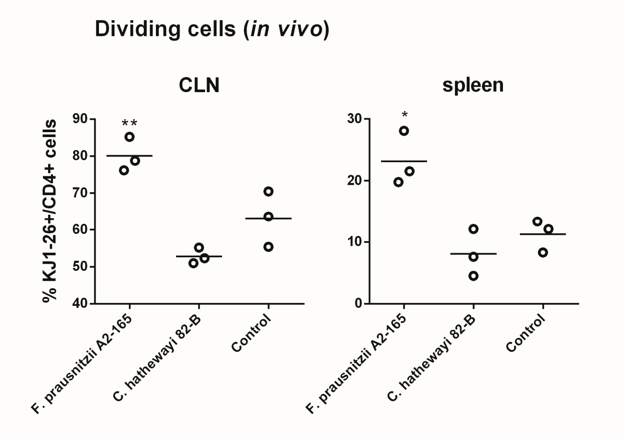

Supplementary Figure S3: Percentage of dividing OVA-T cells *in vivo*. CFSE labeled naive OVA-T cells (KJ1-26^{+}/CD4^{+}) were adoptively transferred in BALB/c mice, after 24 h, mice were administered i.n. with bacteria plus OVA and after additional 72 h, OVA-T cells were isolated from nose-draining CLNs and spleens and analysed. * indicates $p<0.05$, ** indicates $p<0.01$ compared to the control administered OVA alone.

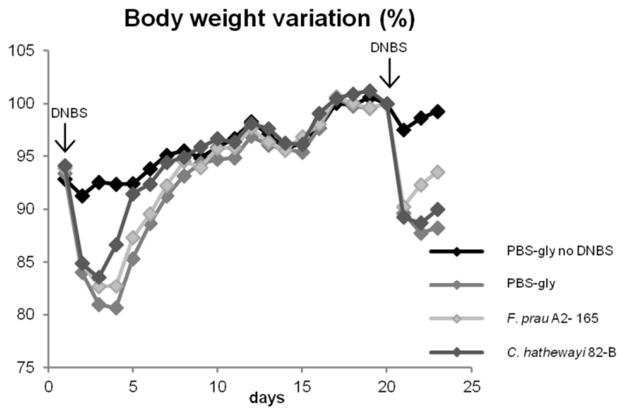

Supplementary Figure S4: Effects of *F. prausnitzii* A2-165 and *C. hathewayi* 82-B on DNBS induced colitis. Weight variation over time in control non-inflamed (PBS-Gly no DNBS), control inflamed (PBS-Gly), *F. prausnitzii* A2-165, *C. hathewayi* 82-B treated mice.

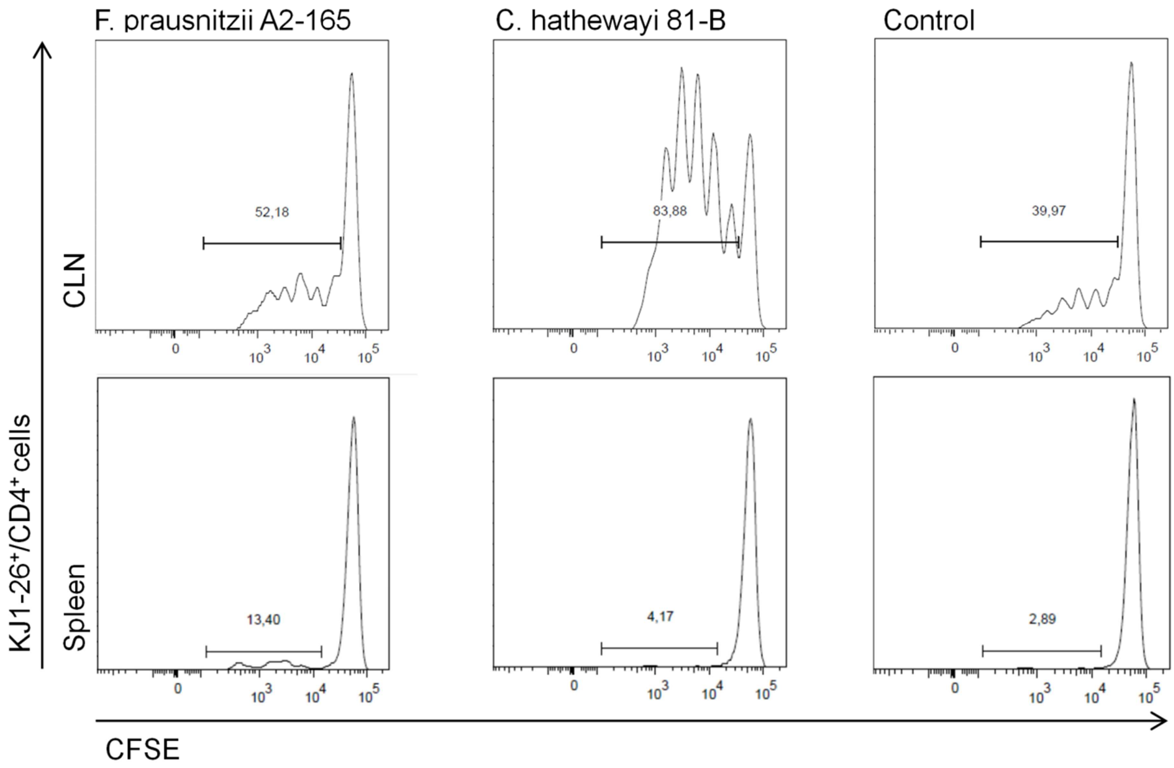

Supplementary Figure S5: Representative histograms of dividing CFSE labelled OVA-T cells *in vivo*. CFSE labelled naive OVA-T cells (KJ1-26$^+$/CD4$^+$) were adoptively transferred in BALB/c mice, after 24 h, mice were administered i.n. with bacteria plus OVA and after additional 72 h, OVA-T cells were isolated from CLNs and spleens and analysed.

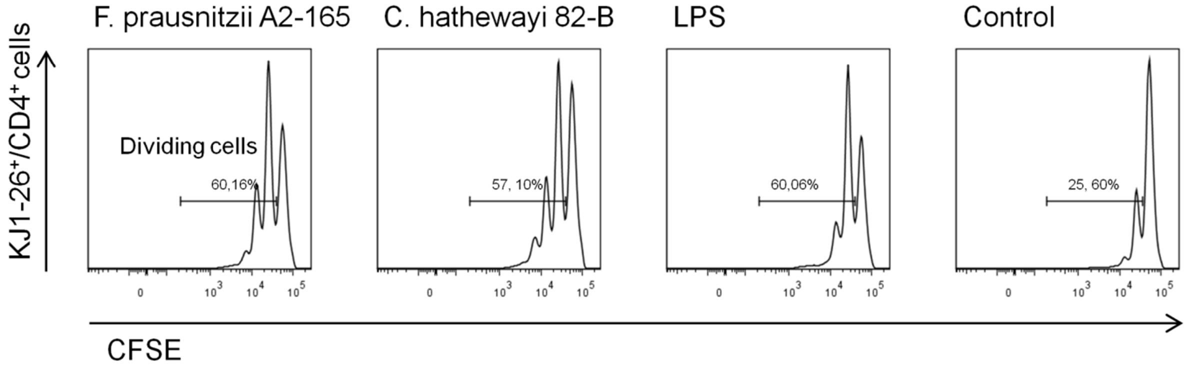

Supplementary Figure S6: Representative histograms of dividing CFSE labelled OVA-T cells after incubation with BMDCs pre-stimulated with bacteria. BMDCs were loaded with OVA and cultured in the presence or absence of the bacteria or LPS and after 24 h, CFSE labelled OVA T cells (KJ1-26^{+}/$CD4^{+}$) were added, after additional 72 h, OVA-T cells were analysed.

Supplementary Methods

TLR signalling assays

TLR assays were performed using human embryonic kidney cells (HEK293) stably expressing human TLR2 and TLR2/6, TLR4 or TLR5 (Invivogen, Toulouse, France) and transfected with a reporter plasmid (pNiFTY, Invivogen) containing the luciferase gene under the control of the NF-κB promoter. HEK293 cells expressing the different TLRs and pNiFTY were seeded at 6 x 10^4 cells/ well in 96-wells plates and incubated with bacteria (bacterium to cell ratio, 10:1), TLR agonists, Pam2CSK4 (Invivogen) for TLR2 and TLR2/6, flagellin (Invivogen) for TLR5 and LPS for TLR4, or medium alone as a control. After 6 h of incubation, the medium was replaced with Bright glow (Promega), and the luminescence was measured using a Spectramax M5 (Molecular Devices). Incubation of each TLR expressing cell line with the respective agonist induced high luciferase activity (Pam2CSK4 is the agonist of TLR2 and TLR2/6, flagellin and LPS are the agonists of TLR5 and TLR4 respectively) compared to cells stimulated with medium alone. As a negative control HEK293 cells not expressing TLRs but harbouring pNiFTY were tested in the same conditions and did not show any luciferase activity. Mean values are shown for 6 replicates, error bars represent SEM.

Human DCs assays

Human immature DCs (hDCs) were differentiated from PBMCs as previously described. After 48 h stimulation of hDCs with bacteria (bacterium to cell ratio, 10:1) or control stimuli the measurement of cytokines in the supernatant and cell surface marker expression was performed as previously described[1].

Mouse BMDCs assays

Bone marrow-cells were isolated by flushing femurs and tibiae of BALB/c mice, and the cell suspension was filtered through 100 µm gauze to obtain single cells. Cells were seeded at $2x10^6$ cells/petri dish in IMDM (Invitrogen) supplemented with 10% heat inactivated FCS, 100U sodium penicillin-G (Bio-Whittaker), 2mM GLutamax (Invitrogen), 50mM 2-mercaptoethanol and 20ng/ml murine rGM-CSF (X63-GM-CSF-producing cell line supernatant[2]) and cultured for 7 days to induce differentiation into DCs. On day 7, cells were seeded at $5x10^5$ cells/well in 24-well plates and were stimulated with bacteria (bacteria to cell ratio, 10:1), 1 µg/ml LPS or left unstimulated. After 24 h the supernatant was collected for cytokine measure and cells were stained for surface protein detection.

Macroscopic scores

Mice were killed by cervical dislocation and the abdominal cavity was opened. The colon was removed and opened longitudinally, macroscopic damage was assessed immediately. Macroscopic scores were recorded using a previously described system for DNBS colitis [3]. Briefly, the macroscopic criteria include macroscopic mucosal damages (such as ulcers, thickening of the colon wall measured by a precision calliper, the presence of adhesions between the colon, and other intra-abdominal organs), the consistency of faecal material (as an indicator of diarrhoea), and the presence of hyperaemia.

Colonic histology and myeloperoxidase activity

Colon samples were fixed in formalin and embedded in paraffin; 3mm-thick sections were cut and stained with Hematoxylin and Eosin (H&E). Colonic damage was evaluated based on a scoring system assessing architectural derangements, goblet cell depletion, edema/ulceration, and degree of inflammatory cell infiltrate. Myeloperoxidase (MPO) activity, was assayed by a modified version of the method previously described [4]. A one-centimetre fragment of the distal colon was recovered and homogenized in ice-cold 50mM potassium phosphate buffer (pH 6) containing 5% hexadecyl trimethyl ammonium bromide (Sigma–Aldrich) and hydrogen peroxide. The colorimetric reaction was followed by measuring the absorbance with a spectrophotometer. MPO activity is expressed as units per milligram of wet tissue.

Purification of DO11.10 T-cells

For the *in vitro* experiments, DO11.10 transgenic T cells were isolated from spleens and lymph nodes of DO11.10 mice and CFSE labelled, as described previously[5,6]. The proportion of DO11.10 transgenic $CD4^+$ T cells in the $CD4^+$ T cell-enriched fraction was determined by flow cytometric analysis of an aliquot of cells using the clonotypic Ab KJ1-26 (anti-OVA transgenic TCR, Caltag) and anti-CD4 (GK1.5, BD). For the *in vivo* adoptive transfer, DO11.10 T cells were isolated from spleens of DO11.10xRag-/- mice, as described above, but the single cell suspension was not enriched.

***In vitro* assays with DO11.10 T cells**

Enriched DO11.10 T cells together with OVA were co-cultured with BMDCs stimulated for 24 h with bacteria. BMDCs were cultured as described above and on day 7, they were seeded at $5x10^4$ cells/well in 96-well plates and stimulated with bacteria (bacteria to cell ratio 10:1), 100 ng/ml LPS or left unstimulated. After 24 h, OVA (Calbiochem) was added to a final

concentration of 0.5 mg/ml and, after additional 6 h, $5x10^5$ CFSE labelled KJ1-26$^+$ CD4$^+$ T cells/well were added. After 72 h, cells were treated with 50 ng/ml Phorbol 12-Myristate 13-Acetate (PMA) and 500 ng/ml Ionomycin to elicit T cell responses; and the intracellular transport of protein was blocked adding monensin (GolgiStop, BD) for the last 4 h of stimulation. Next, the supernatant was collected for cytokine measure and cells were analysed by flow cytometry for cell division and stained for surface and intracellular protein detection.

***Ex vivo* DO11.10 T-cell restimulation**

For *ex vivo* DO11.10 T-cell restimulation, single cells from CLNs (seeded at a density of $3x10^6$ cell/well in 48-wells plates) and from spleens (at a density of 10^7 cells/well in 24-wells plates) of BALB/c acceptor mice were restimulated *in vitro* with 0.2 µg/ml of OVA peptide (OVA 323-339, synthesized and purified by HPLC at the Netherlands Cancer Institute, Amsterdam, the Netherlands). Intracellular transport of protein was blocked adding monensin (GolgiStop, BD) for the last 4.5 h of stimulation. After 24 h, the supernatant was collected for cytokine measure and cells were analysed by flow cytometry for cell division and stained for surface and intracellular protein detection.

Flow cytometry

BMDCs were stained after stimulation with rat anti-mouse CD40 (clone 3/23, BD) followed by donkey anti-rat PE (Jackson), and rat anti-mouse CD86 (BD) and analysed by flow cytometry (FACS Calibur, BD). DO11.10 T cells used *in vitro* and single cells isolated from CLNs and spleens of mice were analysed by flow cytometry to determine their phenotype and division. Cell division was determined based on the decrease of fluorescence intensity of single CFSE peaks. Single cells were stained for surface expression with KJ1-26 (Caltag), anti-CD4 (GK1.5, BD) and for intracellular expression of IL-10 (JES5-16E3), Foxp3 (FJK-16s), IFN-γ (XMG1.2, eBioscience) and IL-17A (TC11-18H10, BD) and their isotype matched control. For flow cytometry analysis, at least $2x10^4$ KJ1-26$^+$/CD4$^+$ cells were used.

References

1 Meijerink, M. *et al.* Identification of genetic loci in Lactobacillus plantarum that modulate the immune response of dendritic cells using comparative genome hybridization. *PloS one* **5**, e10632, doi:10.1371/journal.pone.0010632 (2010).

2 Stockinger, B., Zal, T., Zal, A. & Gray, D. B cells solicit their own help from T cells. *The Journal of experimental medicine* **183**, 891-899 (1996).

3 Qiu, B. S., Vallance, B. A., Blennerhassett, P. A. & Collins, S. M. The role of CD4+ lymphocytes in the susceptibility of mice to stress-induced reactivation of experimental colitis. *Nature medicine* **5**, 1178-1182, doi:10.1038/13503 (1999).

4 Bradley, P. P., Priebat, D. A., Christensen, R. D. & Rothstein, G. Measurement of cutaneous inflammation: estimation of neutrophil content with an enzyme marker. *The Journal of investigative dermatology* **78**, 206-209 (1982).

5 Hauet-Broere, F. *et al.* Functional CD25- and CD25+ mucosal regulatory T cells are induced in gut-draining lymphoid tissue within 48 h after oral antigen application. *European journal of immunology* **33**, 2801-2810, doi:10.1002/eji.200324115 (2003).

6 Unger, W. W. *et al.* Early events in peripheral regulatory T cell induction via the nasal mucosa. *Journal of immunology* **171**, 4592-4603 (2003).
